# Epiphyseal Cartilage Formation Involves Differential Dynamics of Various Cellular Populations During Embryogenesis

**DOI:** 10.3389/fcell.2020.00122

**Published:** 2020-03-05

**Authors:** Yi Zhang, Karl Annusver, Kazunori Sunadome, Polina Kameneva, Steven Edwards, Guanghua Lei, Maria Kasper, Andrei S. Chagin, Igor Adameyko, Meng Xie

**Affiliations:** ^1^Department of Orthopaedics, Xiangya Hospital, Central South University, Changsha, China; ^2^Department of Physiology and Pharmacology, Karolinska Institutet, Solna, Sweden; ^3^Department of Biosciences and Nutrition, Karolinska Institutet, Huddinge, Sweden; ^4^Light Sheet Microscopy Pilot Facility at SciLifeLab, KTH Royal Institute of Technology, Stockholm, Sweden; ^5^Institute for Regenerative Medicine, I.M. Sechenov First Moscow State Medical University, Moscow, Russia; ^6^Department of Molecular Neurosciences, Medical University of Vienna, Vienna, Austria

**Keywords:** joint, cartilage, clonal tracing, asymmetrical, dynamics, embryonic development

## Abstract

A joint connects two or more bones together to form a functional unit that allows different types of bending and movement. Little is known about how the opposing ends of the connected bones are developed. Here, applying various lineage tracing strategies we demonstrate that progenies of Gdf5-, Col2-, Prrx1-, and Gli1-positive cells contribute to the growing epiphyseal cartilage in a spatially asymmetrical manner. In addition, we reveal that cells in the cartilaginous anlagen are likely to be the major sources for epiphyseal cartilage. Moreover, Gli1-positive cells are found to proliferate along the skeletal edges toward the periarticular region of epiphyseal surface. Finally, a switch in the mechanism of growth from cell division to cell influx likely occurs in the epiphyseal cartilage when joint cavitation has completed. Altogether, our findings reveal an asymmetrical mechanism of growth that drives the formation of epiphyseal cartilage ends, which might implicate on how the articular surface of these skeletal elements acquires their unique and sophisticated shape during embryonic development.

## Introduction

Synovial joints are structures located between adjacent skeletal elements, allowing different types of locomotion. The joint surface is covered by articular cartilage and is mechanically stabilized by ligaments that connect the skeletal elements together. Early limb formation starts from a continuous and uninterrupted Y-shaped cartilaginous anlagen (Hamrick, 2001). The developing limb buds consist of undifferentiated mesenchyme that express Paired related homeobox 1 (Prrx1) (Martin and Olson, 2000). The first sign that marks the initiation of the joint formation process is the accumulation of a condensed mesenchymal cell population at the future joint site within the anlagen, so-called the interzone. Cells within the interzone express a new set of genes, including the growth differentiation factor 5 (Gdf5) and serve as joint progenitors by generating a continuous influx of newly produced cells into the developing joint (Koyama et al., 2008; Shwartz et al., 2016). Synovial joints are often uniquely shaped with complex surfaces of adjusted bones matching each other. How this unique morphogenesis is achieved is not clear.

Generally, joint shape morphogenesis begins once the cavitation and synovial space formation initiate (Pacifici et al., 2005), however, one study provided evidence that the process precedes cavitation during hip joint development in chicken (Nowlan and Sharpe, 2014). This remarkable process that defines a variety of distinct joint shapes specific for individual anatomical sites remains the least understood aspect of joint formation. It has been proposed that the proximal and distal ends of the connected skeletal elements might grow simultaneously to complement each other into an interlocking and reciprocally shaped structure (Pacifici et al., 2005). At the cellular level, knee joint development involves cells from the initial anlagen that express collagen type 2 (Col2) and later become interzone cells that turn off Col2 expression, as well as Col2-negative mesenchymal cells recruited from the surroundings that later differentiate into Col2-positive chondrocytes (Hyde et al., 2008).

Hedgehog (Hh) signaling is an evolutionarily conserved pathway that plays important roles in limb development (Tickle and Towers, 2017). There are three Hh homologous proteins in mammals: Sonic hedgehog (Shh), Indian hedgehog (Ihh), and Desert hedgehog (Dhh). When Hh ligands bind to their binding partner Patched (Ptch) at the cell surface it relieves the inhibition of Ptc on another transmembrane protein, Smoothened (Smo) (Corbit et al., 2005; Rohatgi et al., 2007); leading to its phosphorylation by various kinases that eventually activates the Gli transcription factors to promote transcription of Hh target genes (Humke et al., 2010). During embryonic limb development, Shh is expressed in the zone of polarizing activity (ZPA), located in the posterior edge of the limb bud (Wang et al., 2000); where it patterns the anteroposterior axis of the future limb (Kicheva et al., 2012). Chondrocyte-specific overexpression of Shh during murine joint development leads to joint fusion (Tavella et al., 2004). Ihh ligands are important regulators for growth plate chondrocyte proliferation and differentiation (St-Jacques et al., 1999).

In this study, we show that the epiphyseal cartilage grows in an asymmetrical manner that involves differential dynamics of various cellular population.

## Materials and Methods

### Mice

All animal work was permitted by the Ethical Committee on Animal Experiments (Stockholm North committee and Linköping Animal Ethics Committee) and conducted according to The Swedish Animal Agency’s Provisions and Guidelines. Genetic recombination in pregnant female mice was induced by intraperitoneal (ip) injection of tamoxifen (Sigma #T5648). Tamoxifen was dissolved in corn oil at a concentration of 20 mg/ml. 2–5 mg of tamoxifen was injected into the pregnant dam. For plug checking, male and female mice were put together in the evening and vaginal plug was checked early next morning. The day when the plug was observed was considered as E0.5.

The Col2a1-CreERT2 (Nakamura et al., 2006), Prx1-CreERT2 (Kawanami et al., 2009), Gdf5-CreERT2 (Shwartz et al., 2016), Gli1-CreERT2 (Ahn and Joyner, 2004), and R26RConfetti (Snippert et al., 2010) mice have been previously described.

### Chemical Injection

Ethynyl deoxyuridine (Life Technologies #E10187) was dissolved in PBS at a concentration of 1 mg/ml and ip injected into the pregnant dam.

### Epiphyseal Cartilage and Joint Surface Region Definition and Cell Density Quantification

Knee and elbow joint surfaces on images of 2D tissue sections were defined using the ImageJ software. Sagittal sections are collected from about 200 to 300 μm or 100 to 200 μm of the middle regions along the medial-lateral axis, for the knee and elbow joints, respectively. At least, four sections were analyzed per embryo. The ends of epiphyseal cartilage were outlined along the edge of the proximal or distal ends of the four skeletal elements and connected with a straight line between the two ending points of the condyles. The dorsal, middle and ventral regions were defined by equally dividing the connecting straight line into three parts and drawing two straight lines at the dividing points upward to the surface. Number of clones was counted in each region and divided by the total area in mm^2^ to obtain the clonal density. Periarticular region were defined as the cells within the top 50 μm of the epiphyseal cartilage. Length between the dorsal and ventral condylar regions of tibia and radius is defined as the longest distance between the dorsal and ventral sides.

### 3D Analysis of Confetti Clones

3D quantification of the proximal ends of tibia and radius was performed using the Imaris software.

### Tissue Preparation and Immunohistochemistry

Embryos were collected and fixed in 4% PFA for 2–5 h at 4°C. Samples were then washed in PBS at 4°C for 1 h before being placed into 30% sucrose 4°C overnight to eliminate the remaining PFA. Tissues were subsequently embedded in OCT (Tissue-Tek #25608-930) and sectioned into 30–50 μm-thick sections at −20°C. Sections were equilibrated in PBS at room temperature prior to imaging. Primary antibodies (anti-Sox9, Sigma #HPA001758; anti-Col2, Invitrogen #MA5-12789) were applied to the sections for overnight incubation at 4°C. Samples were washed in PBST prior to incubation in secondary fluorescent antibodies for 1 h at room temperature. Images were acquired with an LSM880 confocal microscope.

### Alcian Blue and Eosin Staining

Sections were post fixed in 4% PFA for 5 min at room temperature and then washed with distill water. 0.1% Alcian blue (Sigma #A5268)/0.1 M HCL (Sigma # H1758) solution was applied for 3 min at room temperature, followed by staining in 0.02% eosin for 2 min.

### EDU Retrieval

Frozen sections were used for EdU detection with a click reaction in a mixture of 0.1 M Tris pH 7.5, 2 mM CuSO4, 0.1 M ascorbic acid, and 2 μM Alexa Fluor Azide 647 (Invitrogen #A10277) for 30 min at room temperature, protected from light.

## Results

### Gdf5-Positive Cells Contribute Asymmetrically to the Developing Epiphyseal Cartilage

**FIGURE 1:**
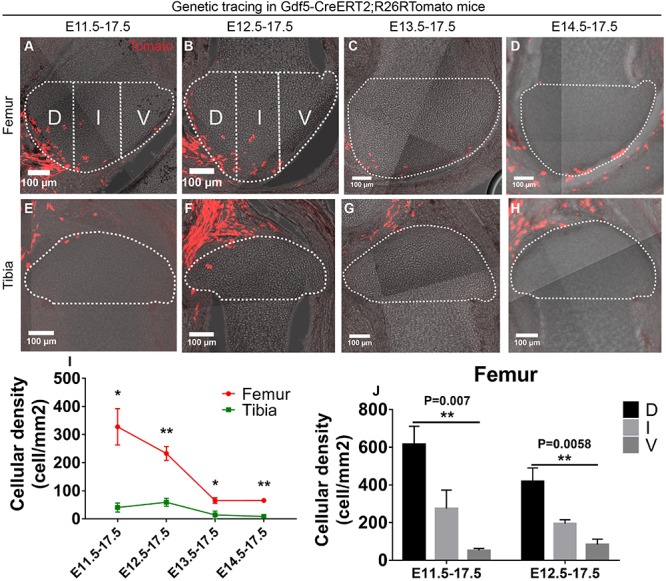
Gdf5-expressing cells contribute asymmetrically to the epiphyseal cartilage development. **(A–H)** Example images showing the distribution of traced Gdf5-expressing cells in the epiphyseal cartilage of knee joint. D, I, and V refer to dorsal condyle, intercondylar eminence and ventral condyle regions, respectively. **(I)** Quantification of the cellular density distribution among the four tracing periods revealed an asymmetrical contribution of the Gdf5-expressing cells to the epiphyseal cartilage of femur and tibia. **(J)** Quantification of the cellular density distribution in the D, I, and V regions during E11.5–E17.5 and E12.5–E17.5 tracings revealed an asymmetrical contribution of the Gdf5-expressing cells to the femoral epiphyseal cartilage along the dorsal-ventral axis. Clonal density represents the number of traced clones over cartilage area (mm^2^). Data represent mean ± SEM, where at least five embryos were analyzed. The white dashed lines outline the epiphyseal cartilage in **(A–H)**. *P < 0.05, **P < 0.01.

Appearance of the interzone at the site of future joint formation marks the initiation of the joint formation process, where the interzone cells start to express a new set of genes, including Gdf5 (Storm et al., 1994; Guo et al., 2004). To investigate how these cells contribute to the developing epiphyseal cartilage ends of the knee and elbow joints during embryogenesis, we used a recently described Gdf5-CreERT2;R26R-Tomato mouse strain that recombines in the interzone mesenchyme (Shwartz et al., 2016). Since joint cavitation takes place between embryonic day (E) 12.5 and E13.5 in the knee and elbow (Supplementary Figure S1), we injected tamoxifen at E11.5, E12.5, E13.5 or E14.5 and collected the samples at E17.5 to reveal the dynamics of Gdf5-positive cells before and after the cavitation process. The overall contribution of the Gdf5-positive cells to the epiphyseal cartilage is rather minor (Figures 1A–H). We noticed that the Gdf5- positive cells specified at all the time points tested, except at E14.5 for the elbow joint, exhibited an uneven distribution pattern between the two opposing ends of the knee and elbow joints; where significantly more Gdf5-traced cells and their progenies were observed in the epiphyseal cartilage of the distal end of femur/humerus compared to the proximal end of tibia/radius (Figures 1A–I and Supplementary Figures S2A–I). In addition, the density of Gdf5-positive cell progenies behaved as a factor of time of tamoxifen injection, which consistently decreased at the distal ends of femur (Figures 1A–D,I) and humerus (Supplementary Figures S2A–D,I) as joint cavitation proceeds; indicating that the interzone cells become a less important player for epiphyseal cartilage construction after cavitation has completed. The density of Gdf5-positive cells at the proximal epiphyseal ends of tibia and radius was too low for any obvious distribution pattern to be reliably detected (Figures 1E–I and Supplementary Figures S2E–I).

We also checked the spatial distribution of the Gdf5-positive cells in the femur and humerus epiphyseal cartilage along the dorsal-ventral axis during E11.5 or E12.5–E17.5 tracings, where the highest amount of Gdf5-positive cells were found in these structures. For the sagittally sectioned samples, we divided the epiphyseal end into three regions of similar size (see section “Materials and Methods”) and referred to as ventral condyle (V), intercondylar eminence (I), and dorsal condyle (D) regions, respectively (Figures 1A,B and Supplementary Figures S2A,B). Then, we quantified the density of Gdf5-positive cells in each region and found that it decreased along the dorsal-ventral or ventral-dorsal axis of the femur and humerus epiphyseal cartilage, respectively, during both tracing periods (Figure 1J and Supplementary Figure S2J). One-day tracing from E12.5 to E13.5 revealed that the labeled cells were mainly present around the joint forming region (Supplementary Figures S2K–N).

Taken together, these results show that the Gdf5-positive cells specified before joint cavitation contribute to the epiphyseal cartilage of knee and elbow joints in an asymmetrical manner both along the longitudinal axis between the two opposing ends and the dorsal-ventral axis within the femur and humerus epiphyseal ends.

### Epiphyseal Cartilage Is Mainly Derived From the Cells Initially Located in the Cartilaginous Anlagen in an Asymmetrical Manner

**FIGURE 2:**
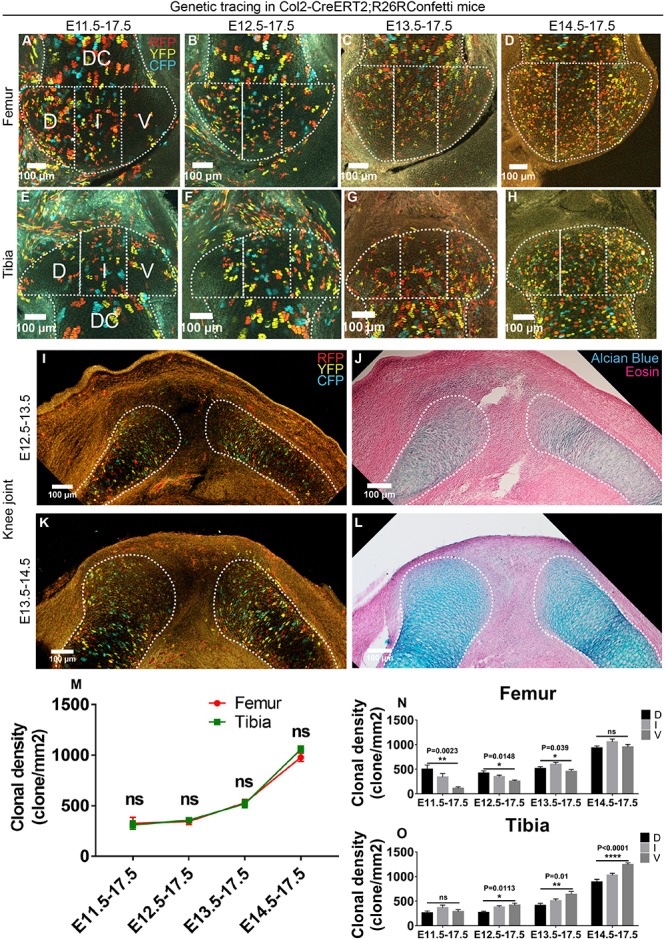
Cells in the cartilaginous anlagen are the major contributors to epiphyseal cartilage development in an asymmetrical manner. **(A–H)** Example images illustrating the distribution of traced Col2-expressing clones in the epiphyseal cartilage of knee joint. D, I, and V refer to dorsal condyle, intercondylar eminence, and ventral condyle regions, respectively. **(I–L)** 1-day tracings of the Col2-expressing cells from E12.5 to E13.5 and E13.5 to E14.5 mainly labeled the cells in the diaphyseal side of the cartilaginous anlagen. **(J)** and **(L)** are alcian blue and eosin staining of the same tissue section on the left. **(M)** Quantification of the clonal density distribution revealed an ascending trend in the epiphyseal cartilage among the four tracing periods. **(N,O)** Quantification of the clonal density distribution in the D, I, and V regions revealed an asymmetrical contribution of the Col2-expressing clones to the femoral and tibial epiphyseal cartilage along the dorsal-ventral axis during many of the analyzed tracing periods. Clonal density represents the number of traced clones over cartilage area (mm^2^). Data represent mean ± SEM, where at least five embryos were analyzed. The white dashed lines outline the epiphyseal and diaphyseal cartilage (DC). *P < 0.05, **P < 0.01, ****P < 0.001, ns, not significant.

The *Col2a1* gene has been shown to be robustly expressed in chondrogenic lineage during early embryonic limb development (Cheah et al., 1985). To label the epiphyseal cartilage progenitors, we then employed the Col2a1-CreERT2 mouse strain that labels the chondrogenic and cartilaginous cells (Nakamura et al., 2006) and coupled it to the R26R-Confetti mouse strain to color-code individual clones (Snippert et al., 2010). The Col2a1-positive clones (referred to as Col2 from here onward) specified at all four time points covered the majority of the epiphyseal cartilage at E17.5 in both the knee (Figures 2A–H) and elbow (Supplementary Figures S3A–H) joints. We also noticed that the later the tracing was initiated the higher overall density of Col2-positive clones was observed in the epiphyseal cartilage, especially when tracing was initiated at E14.5 (Figure 2M and Supplementary Figure S3M). Similar outcome was obtained when analyzing at the 3D level (Supplementary Figures S4A–D). This likely reflects the increase in Col2 expression and suggests maturation of chondrocytes after the completion of joint cavitation.

To examine the cell populations that were labeled by the Col2CreERT2 before and after joint cavitation, we performed 1-day tracings that were initiated at either E12.5 or E13.5. During E12.5–E13.5 tracing, most of the Col2-positive clones were observed in the cartilaginous anlagen of femur, tibia, humerus and radius, especially on the diaphyseal side; and only a few positive clones were observed in the joint-forming region and the surrounding tissues (Figures 2I,J and Supplementary Figures S3I–J). During E13.5–E14.5 tracing, Col2-positive clones were also mainly located in the cartilaginous anlagen, with a more even distribution (Figures 2K,L and Supplementary Figures S3K,L). Since the epiphyseal ends of the knee and elbow joints were largely covered by Col2-positive clones during E12.5 or E13.5–E17.5 tracings (Figures 2B,C,F,G and Supplementary Figures S3B,C,F,G), these results suggest that the epiphyseal cartilage is likely derived from the Col2-positive cells initially located in the cartilaginous anlagen. However, since there is still a few Col2-positive clones labeled outside the anlagen, we cannot completely rule out their roles in epiphyseal cartilage formation.

Unlike the Gdf5-tracings, no difference in clonal density was observed between the two opposing ends of the knee joint and elbow during most of the tracing periods (Figure 2M and Supplementary Figure S3M). We also analyzed the distribution pattern of Col2-positive clones along the dorsal-ventral axis of the epiphyseal ends at both joints. For each skeletal element, the Col2-positive clones exhibited a unique and asymmetrical pattern of distribution during many of the above-mentioned tracing periods (Figures 2N,O and Supplementary Figures S3N,O). In addition, analysis of the entire epiphyseal cartilage at the proximal ends of tibia and radius at the 3D-level demonstrated similar pattern of asymmetrical distribution (Supplementary Figures S4E,F and Supplementary Videos S1–S8).

Taken together, these results show that increasing amount of Col2-positive clones are recruited from the cartilaginous anlagen to the epiphyseal cartilage in an asymmetrical manner along the dorsal-ventral axis.

### Prrx1 Expression Is Down Regulated in Epiphyseal Cartilage Progenitors as Joint Cavitation Proceeds

**FIGURE 3:**
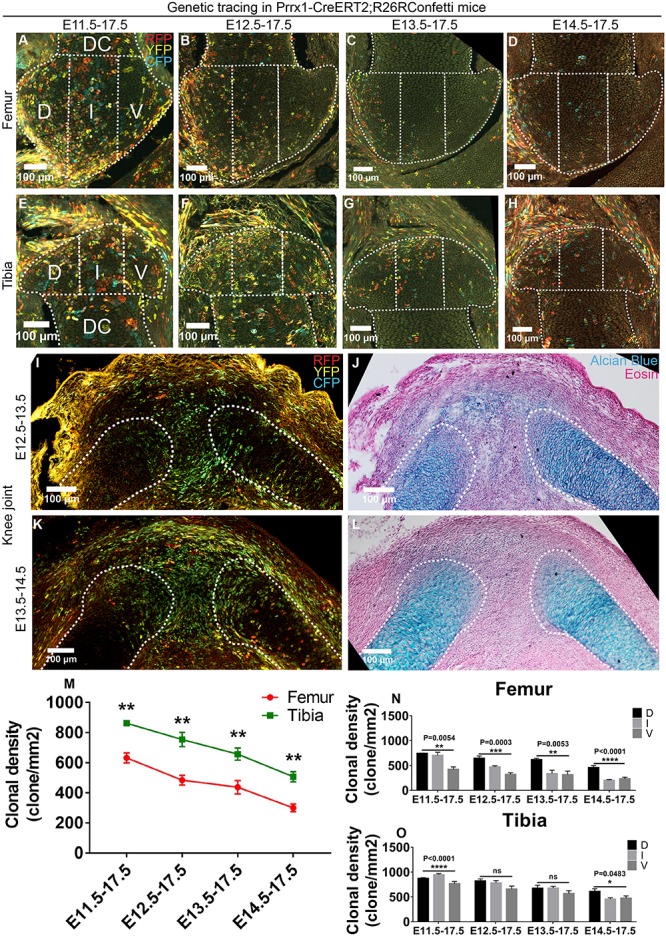
Prrx1 expression decreases in epiphyseal progenitors as joint cavitation proceeds. **(A–H)** Example images illustrating the distribution of traced Prrx1-expressing clones in the epiphyseal cartilage of knee joint. D, I, and V refer to dorsal condyle, intercondylar eminence, and ventral condyle regions, respectively. **(I–L)** 1-day tracings of the Prrx1-expressing cells from E12.5 to E13.5 and E13.5 to E14.5 mainly labeled the cells in the joint forming region and epiphyseal side of the cartilaginous anlagen. **(J)** and **(L)** are alcian blue and eosin staining of the same tissue section on the left. **(M)** Quantification of the clonal density distribution revealed a descending trend in the epiphyseal cartilage among the four tracing periods and an asymmetrical contribution to the femoral and tibial epiphyseal ends. **(N,O)** Quantification of the clonal density distribution in the D, I, and V regions revealed an asymmetrical contribution of the Prrx1-expressing clones to the femoral and tibial epiphyseal cartilage along the dorsal-ventral axis during many of the analyzed tracing periods. Clonal density represents the number of traced clones over cartilage area (mm^2^). Data represent mean ± SEM, where at least five embryos were analyzed. The white dashed lines outline the epiphyseal and diaphyseal cartilage (DC). *P < 0.05, **P < 0.01, ***P < 0.005, ****P < 0.001, ns, not significant.

After checking the contribution of interzone and chondrogenic cells, we then wondered how the earlier cell populations, i.e., the limb mesenchymal cells, contribute to the developing epiphyseal cartilage. Therefore, we employed the Prrx1-CreERT2 mouse strain to label the mesenchymal progenitor cells of the limb bud (Kawanami et al., 2009) and coupled it with the R26R-Confetti strain. Contrary to the Col2-tracings, a reversed trend in the stage-dependent clonal density distribution was observed for the Prrx1-positive clones, i.e., the later the tracing started, the less Prrx1-positive clones were observed in the epiphyseal cartilage of both the knee (Figures 3A–H,M) and elbow joints (Supplementary Figures S5A–H,M). This suggests that Prrx1 expression is down-regulated in the epiphyseal progenitors as joint cavitation proceeds, likely correlates with the differentiation of these cells into mature chondrocytes.

We also performed 1-day tracings to find out the cell populations that were actually labeled in the knee and elbow joints before (E12.5) and after (E13.5) cavitation. During both tracings, Prrx1-CreERT2 labeled most of the cells in the joint forming region and the epiphysis of the cartilaginous anlagen, as well as some of the chondrocytes in the diaphysis (Figures 3I–L and Supplementary Figures S5I–L). Considering the diminished role of Prrx1-positive cells at the epiphyseal ends as joint cavitation proceeds (Figure 3M and Supplementary Figure S5M), it is likely that the intensively labeled Prrx1-positive cells in the joint cavity and the surroundings only make minor contribution to the epiphyseal cartilage. The Prrx1-positive cells in the cartilaginous anlagen are likely the same population of the Col2-positive cells that give rise to the epiphyseal cartilage.

Unlike the Col2-tracings, significantly more Prrx1-positive clones were observed in the tibial epiphyseal cartilage compared to the femoral side during all tracing periods (Figure 3M), which is completely opposite to the distribution pattern of the Gdf5-traced cells between the two ends (Figure 1I). Intriguingly, such difference was not detected at the elbow joint (Supplementary Figure S5M). In addition, asymmetrical distribution of the Prrx1-positive clones was also observed along the dorsal-ventral axis of the epiphyseal cartilage of both the knee and elbow joints during many of the tracing periods (Figures 3N,O and Supplementary Figures S5N,O). Such spatial differences in the distribution pattern of Col2- and Prrx1-positive clones were not observed in the developing diaphyseal cartilage during all four tracing periods (Supplementary Figure S6), indicating that construction of the epiphyseal and diaphyseal cartilage involves differential cellular dynamics.

Taken together, these results further suggest that the epiphyseal cartilage are not formed via an even cellular contribution, but rather employs an asymmetrical mechanism of growth along the longitudinal and dorsal-ventral axes.

### Epiphyseal Cartilage Formation After Joint Cavitation Involves Newly Recruited Col2-Positive Clones

**FIGURE 4:**
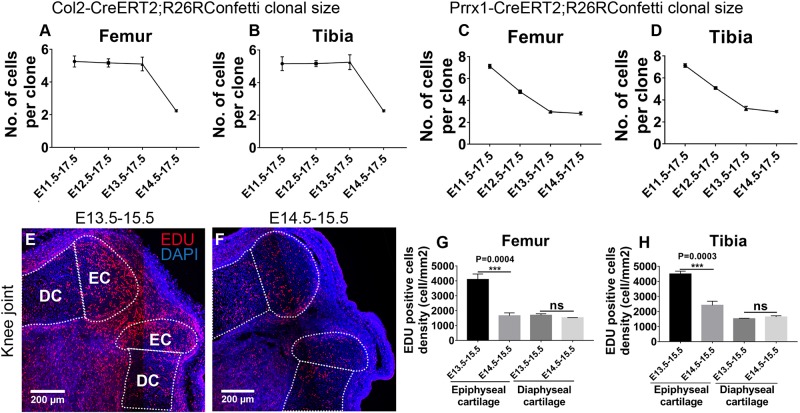
Epiphyseal cartilage formation involves influx of Col2-expressing cells post joint cavitation. **(A,B)** Size of the traced Col2-expressing clones was significantly reduced in the femoral and tibial epiphyseal cartilage during E14.5–E17.5 tracing. **(C,D)** Size of the traced Prrx1-expressing clones gradually decreased as the tracing was initiated at later stages. **(E,F)** Example EDU staining images of knee joint in wild type mice injected with EDU at E13.5 or E14.5. **(G,H)** More EDU-positive cells were found in the epiphyseal cartilage, but not the underneath growth plate, of both femur and tibia when EDU was injected at E13.5 compared to injection at E14.5. EDU-positive cell density represents the number of EDU-positive cells over cartilage area (mm^2^). Data represent mean ± SEM, where at least three embryos were analyzed. The white dashed lines outline the epiphyseal cartilage (EC) and diaphyseal cartilage (DC). ***P < 0.005, ns, not significant.

Color-coding of individual clones with the R26R-Confetti reporter allows us to quantify the number of cells in each Col2- and Prrx1-positive clone and analyze the clonal dynamics based on their size distribution among all tracing periods in the epiphyseal cartilage. At the knee joint, the average size of Col2-positive clones remained at about five cells per clone during E11.5, E12.5 or E13.5–E17.5 tracings; however, it dropped sharply to only two cells per clone when tracing was initiated at E14.5 (Figures 4A,B). This is opposite to the trend of Col2-positive clonal density, where a sharp increase was observed when traced at E14.5 (Figure 2M). To confirm that the sharp reduction in clonal size traced at E14.5 was a result of reduced cell division, we assessed the proliferation status of the epiphyseal cells by injecting ethynyl deoxyuridine (EDU) at E13.5 or E14.5 and collected at E15.5 for analysis. Indeed, significantly more EDU-positive cells were observed in the knee and elbow epiphyseal cartilage, but not the diaphyseal cartilage, of the E13.5 injected embryos than the E14.5 injected ones (Figures 4E–H and Supplementary Figures S7E–H); further suggesting that the reduced clonal size is a result of decreased cell division. Therefore, proliferation of the epiphyseal cartilage is likely not a result of even proliferation of chondrocytes, in which case both the clonal density and size would simply be bigger for animals injected at earlier stages. On the other hand, these observations can be explained by the *de novo* appearance of Col2-positive clones that are formed after joint cavitation, i.e., Col2-positive cells that are labeled at E14.5.

Taken together, when labeled at E11.5, E12.5 or E13.5, density of the Col2-positive clones steadily increases while the clonal size remains consistent, suggesting a continuous mechanism of epiphyseal cartilage development as joint cavitation proceeds. At E14.5, an increased amount of Col2-positive cells are recruited to the epiphyseal cartilage, where they divide much less actively than the ones specified earlier; indicating that the mechanism of epiphyseal cartilage formation may switch from cell division to cell influx when joint cavitation has completed.

For the Prrx1-positive clones, the average clonal size steadily decreased from approximately seven cells per clone to three cells per clone in a two-cell interval during E11.5, E12.5 or E13.5–E17.5 tracings and remained at the same size during E14.5–E17.5 tracing (Figures 4C,D), suggesting that the Prrx1-positive cells specified before joint cavitation divide at a relatively constant rate, whereas the ones specified after joint cavitation divide much less actively. Similar trends of clonal size distribution were observed at the elbow joints for both Col2- and Prrx1-positive clones with an overall slightly more smooth decrease compared to the corresponding knee joints (Supplementary Figures S7A–D).

### Gli1-Positive Cells Proliferate Along the Skeletal Edges to Cover the Periarticular Region of Epiphyseal Surface

**FIGURE 5:**
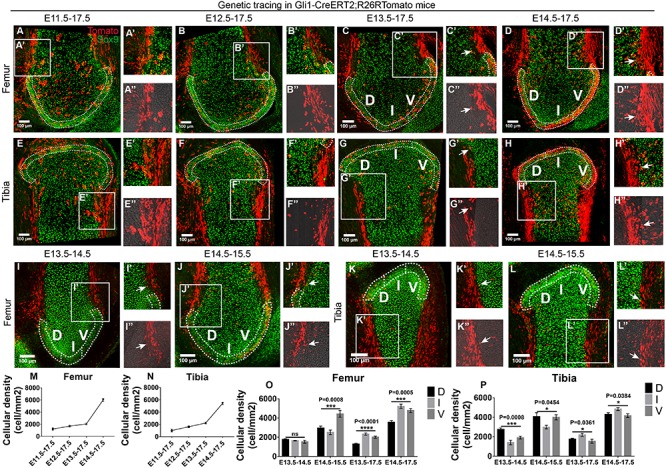
Gli1-expressing cells along the skeletal edges give rise to the periarticular region of epiphyseal surface. **(A–L)** Example images of lineage tracings in Gli1-CreERT2;R26RTomato mice at the knee joint surface. Sox9 staining was added to help define the cartilage. **(M,N)** Increased density of Gli1-positive cells were found in the periarticular region when tracing was started at later time points. **(O,P)** Gli1-positive cells proliferate from the dorsal (D) and ventral (V) edges of the epiphyseal surface toward the intercondylar eminence (I) when cavitation has completed. Data represent mean ± SEM where at least four embryos were analyzed. The white dashed lines outline the periarticular region of the epiphyseal surface. Arrows in the insets of **(C,D,G–L)** point to the Tomato and Sox9 double positive perichondrial cells. Insets labeled with double apostrophe marks show Tomato only **(A–L)**. *P < 0.05, ***P < 0.005, ****P < 0.001, ns, not significant.

Next, we sought to explore the signaling pathways and responsive cell types that govern the formation of epiphyseal cartilage. Given that Hh acts as a major morphogen in limb bud and cartilage patterning during murine embryonic development (Wang et al., 2000; Kicheva et al., 2012), we obtained the Gli1-CreERT2;R26R-Tomato mouse strain that labels Hh-responsive cells upon tamoxifen injection (Ahn and Joyner, 2004) to check their tracing patterns during the four above-mentioned tracing periods at the epiphyseal ends of the knee and elbow joints. At the knee joint, we found that the surrounding cells along the dorsal and ventral edges of both skeletal elements and some perichondrial cells were consistently labeled in all conditions (Figures 5A–H). In addition, the density of Gli1-positive cells in the periarticular region of the epiphyseal surface [defined as the 4–6 layers of chondrocytes within the top 50 μm of the epiphyseal surface (Tong et al., 2019)] was significantly increased when tracing was initiated at later time points, especially at E13.5 and E14.5 where majority of the periarticular region was covered by Gli1-positive cells (Figures 5A–H,M,N). These results suggest that the Hh-responsive cells specified after joint cavitation are the major contributors to the periarticular region of the knee joint. Chondrocytes were counterstained with Sox9 to better define the cartilage (Ng et al., 1997; Zhao et al., 1997).

To further dissect the cellular mechanism underlying the formation of the periarticular region, we performed 1-day tracing from E13.5 to E14.5 and E14.5 to E15.5 to assess the initial location of the Gli1-positive cells. Employing the same spatial analysis strategy for the epiphyseal cartilage, we divided the periarticular region into three sub-regions, i.e., ventral condyle (V), intercondylar eminence (I), and dorsal condyle (D), to quantify the density of Gli1-positive cells in each sub-region (Figures 5I–L). With 1-day tracing starting from either E13.5 or E14.5, more Gli1-positive cells were found in the V and D sub-regions compared to the I sub-region (Figures 5O,P). However, with longer tracing until E17.5, more Gli1-positive cells were found in the I sub-region, compared to the other two (Figures 5O,P). In addition, the overall density of Gli1-positive cells within the periarticular region was much higher with tracings started from E14.5, compared to tracings started from E13.5 (Figures 5O,P). These results suggest that chondrocytes within the periarticular region are mostly derived from the Gli1-positive cells along the dorsal and ventral edges of the femur and tibia, where they likely proliferate along the edges toward the plateau to eventually cover the entire periarticular region. When tracing from E15.5 to E17.5, significantly less periarticular cells were labeled compared to the E14.5–E17.5 tracing (Supplementary Figure S9), suggesting that these cells respond less to the Hh signaling from E15.5.

Intriguingly, tracing pattern of the Gli1-positive cells at the elbow joint appeared to be rather random. Surrounding and perichondrial cells were not consistently labeled (Supplementary Figures S8A–L) and the density of Gli1-positive cells in the periarticular region varies among the four tracing periods (Supplementary Figures S8M,N). No obvious distribution pattern was recognized among the three sub-regions either (Supplementary Figures S8O,P). This suggests that the Gli1-positive cells may have differential roles in the development of elbow and knee epiphyseal surface.

## Discussion

Previous study using Col2-Cre;R26R mice showed that the Col2-positive anlagen proliferate until the presence of interzone cells at the future joint site, where they no longer express Col2 and start to express Gdf5 (Hyde et al., 2008). In addition, a continuous influx of Gdf5-positive cells has been shown to contribute to the formation of various joint structures, including the articular surface where they start to express Col2 again and become mature chondrocytes (Shwartz et al., 2016). Using various lineage tracing strategies, our work builds on top of the existing influx model of joint development to quantitatively demonstrate the asymmetrical engagement of progenies of the Gdf5-, Col2-, Prrx1-, and Gli1-positive cells into the generation of new cartilage at the epiphyseal ends.

**FIGURE 6:**
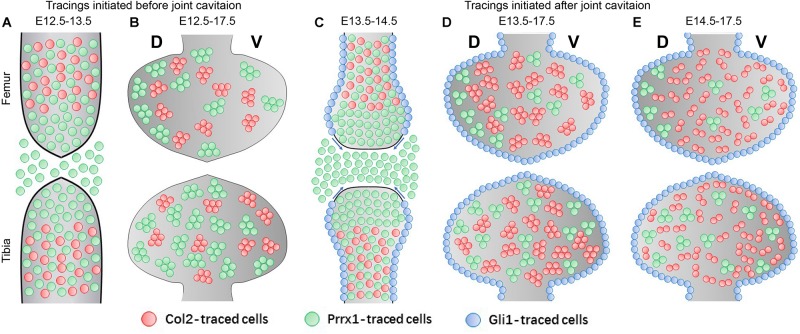
A model for epiphyseal cartilage development at the knee joint surface. **(A)** When labeled before joint cavitation, Col2 is mainly expressed in the cells of the diaphyseal part of the cartilaginous anlagen, whereas Prrx1 is expressed in cells located in the joint forming region, the epiphyseal part of the cartilaginous anlagen and the surround tissues, as well as the diaphyseal anlagen. **(B)** Both the traced Col2- and Prrx1-positive clones occupy majority of the epiphyseal cartilage at E17.5. **(C)** When labeled after joint cavitation, Col2 is mainly expressed in the cells of the cartilaginous anlagen, whereas Prrx1-expressing cells mark almost the entire joint forming region and the epiphyseal anlagen. At the meantime, Gli1-expressing cells lineup along the dorsal and ventral edge of the anlagen, likely migrating toward the periarticular region of epiphyseal surface. **(D,E)** The epiphyseal cartilage is almost entirely composed of the Col2-traced clones at E17.5, whereas the Prrx1-traced clones are found at a much lesser extent. New Col2-expressing cells are recruited to the epiphyseal cartilage when labeled at E14.5 as reflected by the significant drop in their clonal size. Meanwhile, the Gli1-traced cells cover the entire periarticular region during this time. The asymmetrical distribution of the Col2- and Prrx1-traced clones along both the longitudinal axis between the two opposing ends and the dorsal-ventral axis within each skeletal element is also reflected in **(B,D,E)**.

When labeled before joint cavitation at E12.5, Col2-positive cells were mainly present in the cartilaginous anlagen toward the diaphyseal side; whereas Prrx1-positive cells occupied a decent fraction of the joint forming region and the epiphyseal side of the anlagen, as well as part of the diaphysis (Figure 6A). At E17.5, the developing epiphyseal cartilage is largely covered by both the Col2- and the Prrx1-positive clones of similar size in an asymmetrical manner (Figure 6B). Therefore, the epiphyseal cartilage is likely constructed by an overlapping population of Col2- and Prrx1-positive cells specified before joint cavitation. Based on the 1-day tracing pattern of both strains, the only major overlapping population is the cells located on the diaphyseal side of the anlagen, hinting the cellular origin of the epiphyseal cartilage. When labeled after joint cavitation at E13.5, Col2-positive cells were still mainly located in the cartilaginous anlagen, whereas Prrx1 was expressed in almost all cells of the joint forming region and the epiphyseal side of the anlagen, but only in some of the diaphyseal cells (Figure 6C). Meanwhile, Gli1-positive cells were observed along the edges of the anlagen, likely proliferating toward the periarticular region of epiphyseal surface (Figure 6C). At E17.5, the developing cartilage was mainly occupied by the Col2-positive clones, but to a much lesser extent by the Prrx1-positive clones; and the entire periarticular region was covered by the Gli1-positive cells (Figure 6D). This further excludes the Prrx1-positive cells in the joint forming region and the epiphyseal side of the anlagen as the progenitors for epiphyseal cartilage growth, in which case their density should be increased or maintained at E17.5. Thus, cells in the cartilaginous anlagen might be the major cellular source for epiphyseal cartilage growth. At E14.5, the major developing mechanism of epiphyseal cartilage likely switches from cell division to cell influx, where the traced Col2-positive clones becomes significantly smaller in size, accompanied by a dramatic increase in density (Figure 6E); although we cannot fully exclude the influence from the difference in the length of tracing periods.

It is worth noting that the mouse lines used in this study likely label overlapping cell populations, although each strain has a relatively unique labeling pattern based on the 1-day tracings. Therefore, the contribution patterns observed for different lineage tracings likely involve overlapping cellular populations, especially for the labeled cells in the cartilaginous anlagen, many of which are positive for both Col2 and Prrx1. We cannot completely exclude the effects of tamoxifen administration and distribution, CreERT2 efficiency and transgene nature and expression on the outcome of the tracing experiments. However, based on the stable clonal size and unique clonal distribution observed in different embryos for each tracing period, as well as the decent Cre recombination efficiency in all tested strains as reflected by the amount of labeled cells; these technical issues should not produce any profound interference on our conclusions.

Previous studies show that the Gdf5-positive cells contribute to joint formation in a temporally different manner, where the early specified Gdf5-positive cells make the most contribution to the epiphysis (Storm and Kingsley, 1999; Shwartz et al., 2016). Consistently, we also observed a decreasing trend in Gdf5-positive cell density in the epiphyseal cartilage along the induction time line. It is worth noting that although the overall level of Gdf5-positive cell in the epiphyseal cartilage is relatively low, it does not truly reflect the recombination efficiency of Gdf5CreERT2. Our recent findings show that the cruciate ligament is highly labeled in the same strain, especially during the E11.5–E17.5 and E12.5–E17.5 tracings (Zhang et al., 2020). On top, our data indicate that from E12.5 onward the Col2-positive cells in the cartilaginous anlagen likely become the major contributors to the epiphyseal cartilage. A temporal switch in the major mechanism of epiphyseal cartilage formation from influx of the Gdf5-positive cells to proliferation of the Col2-positive cells might exist as cells in the anlagen become mature chondrocytes. Our observation is in line with another previous study using EDU and BrdU incorporation methods to show that the embryonic articular cartilage cells originate from the proliferating chondrocytes located in the distal anlagen (Ray et al., 2015).

Early limb develops from a single piece of uninterrupted mesenchymal condensation, which later goes through a cavitation process to physically separate into zeugopod and stylopod. Current understanding of joint cavitation believes that cell death plays a major role in the separation of the continuous skeletal anlagen (Mitrovic, 1978; Nalin et al., 1995; Kimura and Shiota, 1996; Abu-Hijleh et al., 1997), albeit it has been challenged by other studies (Ito and Kida, 2000). Gli1 has recently been identified as a marker for osteogenic progenitors in murine long bone formation (Shi et al., 2017) and for mesenchymal stem cells within the suture mesenchyme for craniofacial bone development (Zhao et al., 2015). Constitutive activation of Hh signaling specifically in the interzone cells caused severe morphological changes in murine knee joint (Rockel et al., 2016). Overexpression of Shh in chondrocytes abolished the joint cavity due to disrupted cell apoptosis and proliferation (Tavella et al., 2004). Our observation that the Gli1-positive cells labeled at E13.5 or E14.5, time points when joint cavitation has just accomplished, are mainly present along the edges of the developing femur and tibia and proliferate toward the periarticular region of epiphyseal surface that eventually give rise to the entire structures further hints a potential role of these cells in the cavitation process. Convergence of the Gli1-positive cells from the two sides might mark the end point of the joint cavitation process. Influx of the surrounding cells might be an additional source for periarticular cartilage formation since some of them also express Gli1 during the tracings. This complies with the previous finding that articular cartilage continually express Gdf5 during multiple embryonic tracings (Shwartz et al., 2016).

Shape of the epiphyseal cartilage is one of the main factors that determines joint stability. The architecture of joint shape is extensively related to the pathogenesis of osteoarthritis. Many studies have suggested that tibial and femoral bone morphology at the knee joint is a risk factor for anterior cruciate ligament (ACL) injury (Lansdown and Ma, 2018; Vasta et al., 2018) and medial meniscus tears (Barber et al., 2017), especially the tibial plateau slope (Wang et al., 2017). Not only to the susceptibility to ACL injury, post trauma recovery is also linked to the bone shape differences (Lansdown et al., 2017). In the present study, we analyzed the spatial distribution, density and size of Gdf5-, Col2-, Prrx1-, and Gli1-positive cells/clones to investigate how the epiphyseal cartilage is developed when recombination is induced at different time points, which can be cross-compared. It seems that epiphyseal cartilage does not proliferate via continuous expansion of the pre-existing chondrocyte clones, in which case the clonal density and size would simply be bigger for embryos injected at earlier stages. Alternatively, increasing amount of Col2-positive chondrogenic clones are asymmetrically recruited to the epiphyseal cartilage at a relatively late time point, i.e., post joint cavitation. These clones are small in term of clonal size and stable in term of clonal geometry, most of which contain only two cells. Our data support and extend the previously established cell influx model for joint development (Shwartz et al., 2016) by showing how many chondrogenic cells are engaged into generation of new cartilage in the epiphyseal cartilage and how asymmetrically they are distributed during this process. Our data suggest that the proliferation of pre-existing cartilage is limited and rather uniform, whereas the asymmetrical influx and proliferation of pro-chondrogenic cells likely accounts for the asymmetrically developed epiphyseal ends. Such a temporally (before and after joint cavitation) and spatially (along the dorsal-ventral and longitudinal axes) asymmetrical growth mechanism of epiphyseal cartilage might provide indications on the cellular dynamics underlying the development of various shaping features of the joint ends. In addition, we uncover the differential contribution of several known cellular sources for the epiphyseal cartilage during embryonic development, which might impinge on the cellular and molecular target selection for the development of novel treatment against clinical conditions as a result of abnormal fetal joint morphogenesis, such as hip dysplasia.

## Data Availability Statement

The datasets generated for this study are available on request to the corresponding author.

## Ethics Statement

The animal study was reviewed and approved by the Stockholm North or Linköping Ethical Committee.

## Author Contributions

MX, IA, and YZ designed the study. YZ performed all the experiments, except those specified below. KA and MK provided the Gli1-CreERT2;R26R-Tomato mouse strain and performed the genetic tracings and embryo collection. PK and MX helped with mouse embryonic tracing experiments. SE helped with light sheet microscopic imaging for 3D clonal analysis. AC, KS, and GL provided intellectual input into the manuscript. MX and IA wrote the manuscript.

## Conflict of Interest

The authors declare that the research was conducted in the absence of any commercial or financial relationships that could be construed as a potential conflict of interest.

## Supplementary Material

FIGURE S1 Alcian blue **(A–D)**, Col2 and Sox9 **(E–P)** staining of the knee and elbow joint during at E12.5 and E13.5. The white dashed lines outline the cartilage.

FIGURE S2 **(A–H)** Example images showing the distribution of traced Gdf5-expressing cells in the epiphyseal cartilage of elbow joint. D, I, and V refer to dorsal condyle, intercondylar eminence, and ventral condyle regions, respectively. **(I)** Quantification of the cellular density distribution among the four tracing periods revealed an asymmetrical contribution of the Gdf5-expressing cells to the epiphyseal cartilage of humerus and radius. **(J)** Quantification of the cellular density distribution in the D, I, and V regions during E11.5–E17.5 and E12.5–E17.5 tracings revealed an asymmetrical contribution of the Gdf5-expressing cells to the humerus epiphyseal cartilage along the dorsal-ventral axis. **(K**–**N)** 1-day tracings of the Gdf5-expressing cells from E12.5 to E13.5 in knee **(K,L)** and elbow **(M,N)** joints mainly labeled the cells around the joint forming region. **(L)** and **(N)** are alcian blue and eosin staining of the same tissue section on the left. Clonal density represents the number of traced clones over cartilage area (mm^2^). Data represent mean ± SEM, where at least five embryos were analyzed. The white dashed lines outline the epiphyseal cartilage. ***P* < 0.01, ****P* < 0.005, *****P* < 0.001.

FIGURE S3 **(A–H)** Example images illustrating the distribution of traced Col2-expressing clones in the epiphyseal cartilage of elbow joint. D, I, and V refer to dorsal condyle, intercondylar eminence and ventral condyle regions, respectively. **(I–L)** 1-day tracings of the Col2-expressing cells from E12.5 to E13.5 and E13.5 to E14.5 mainly labeled the cells in the diaphyseal side of the cartilaginous anlagen. **(J)** and **(L)** are alcian blue and eosin staining of the same tissue section on the left. **(M)** Quantification of the clonal density distribution revealed an ascending trend in the epiphyseal cartilage among the four tracing periods. **(N,O)** Quantification of the clonal density distribution in the D, I, and V regions revealed an asymmetrical contribution of the Col2-expressing clones to the humerus and radius epiphyseal cartilage along the dorsal-ventral axis during many of the analyzed tracing periods. Clonal density represents the number of traced clones over cartilage area (mm^2^). Data represent mean ± SEM, where at least five embryos were analyzed. The white dashed lines outline the epiphyseal and diaphyseal cartilage (DC). **P* < 0.05, ***P* < 0.01, ****P* < 0.005, *****P* < 0.001.

FIGURE S4 **(A–D)** 3D analysis of traced Col2-expressing clonal density in the entire epiphyseal cartilage of the four skeletal elements. **(E,F)** Distribution of traced Col2-expressing clones in dorsal condyle (D), intercondylar eminence (I), and ventral condyle (V) regions of the entire tibia and radius epiphyseal cartilage at the 3D level. Clonal density represents the number of traced clones over cartilage area (mm^2^). Data represent mean ± SEM, where at least five embryos were analyzed. **P* < 0.05, ***P* < 0.01.

FIGURE S5 **(A–H)** Example images illustrating the distribution of traced Prrx1-expressing clones in the epiphyseal cartilage of elbow joint. D, I, and V refer to dorsal condyle, intercondylar eminence and ventral condyle regions, respectively. **(I–L)** 1-day tracings of the Prrx1-expressing cells from E12.5 to E13.5 and E13.5 to E14.5 mainly labeled the cells in the joint forming region and epiphyseal side of the cartilaginous anlagen. **(J)** and **(L)** are alcian blue and eosin staining of the same tissue section on the left. **(M)** Quantification of the clonal density distribution revealed a descending trend in the epiphyseal cartilage among the four tracing periods. **(N,O)** Quantification of the clonal density distribution in the D, I, and V regions revealed an asymmetrical contribution of the Prrx1-expressing clones to the humerus and radius epiphyseal cartilage along the dorsal-ventral axis during many of the analyzed tracing periods. Clonal density represents the number of traced clones over cartilage area (mm^2^). Data represent mean ± SEM, where at least five embryos were analyzed. The white dashed lines outline the epiphyseal and diaphyseal cartilage (DC). **P* < 0.05.

FIGURE S6 **(A–H)** The Col2- and Prrx1-traced clones distribute evenly in the dorsal condyle (D), intercondylar eminence (I), and ventral condyle (V) regions of the diaphyseal cartilage of the four skeletal elements. Clonal density represents the number of traced clones over cartilage area (mm^2^). Data represent mean ± SEM, where at least five embryos were analyzed.

FIGURE S7 **(A,B)** Size of the traced Col2-expressing clones dropped sharply in the humerus and radius epiphyseal cartilage during E14.5–E17.5 tracing. **(C,D)** Size of the traced Prrx1-expressing clones gradually decreased as the tracing was initiated at later stages. **(E,F)** Example EDU staining images of elbow joint in wild type mice injected with EDU at E13.5 or E14.5. **(G,H)** More EDU-positive cells were found in the epiphyseal cartilage, but not the underneath growth plate, of both humerus and radius when EDU was injected at E13.5 compared to injection at E14.5. EDU-positive cell density represents the number of EDU-positive cells over cartilage area (mm^2^). Data represent mean ± SEM, where at least three embryos were analyzed. The white dashed lines outline the epiphyseal cartilage (EC) and diaphyseal cartilage (DC). ****P* < 0.005, *****P* < 0.001.

FIGURE S8 **(A–L)** Example images of lineage tracings in Gli1-CreERT2;R26RTomato mice at the elbow joint surface. **(M–P)** No obvious pattern was observed for Gli1-tracings at the elbow joint periarticular region. Data represent mean ± SEM where at least four embryos were analyzed. The white dashed lines outline the periarticular region of epiphyseal surface. D, I, and V refer to dorsal condyle, intercondylar eminence and ventral condyle sub-regions, respectively. **P* < 0.05, ***P* < 0.01.

FIGURE S9 **(A–H)** Gli1-positive cell density dropped sharply in the periarticular region of epiphyseal surface of knee and elbow joint during E15.5–E17.5 tracing compared to E14.5–E17.5 tracing. Data represent mean ± SEM where at least four embryos were analyzed. The white dashed lines outline the periarticular region of epiphyseal surface. ****P* < 0.005, *****P* < 0.001.

VIDEOS S1–S8 3D scans of the knee (S1, S3, S5, S7) and elbow (S2, S4, S6, S8) joints of Col2-CreERT2;R26RConfetti mice traced from E11.5, E12.5, E13.5 or E14.5 to E17.5. The upper skeletal elements are femur or humerus and the lower ones are tibia or radius in all videos.
