# Supplementary figures and images for: Epiphyseal Cartilage Formation Involves Differential Dynamics of Various Cellular Populations During Embryogenesis
Source: Front Cell Dev Biol. 2020 Mar 5;8:122. doi: 10.3389/fcell.2020.00122 (PMC7066500; doi:10.3389/fcell.2020.00122)

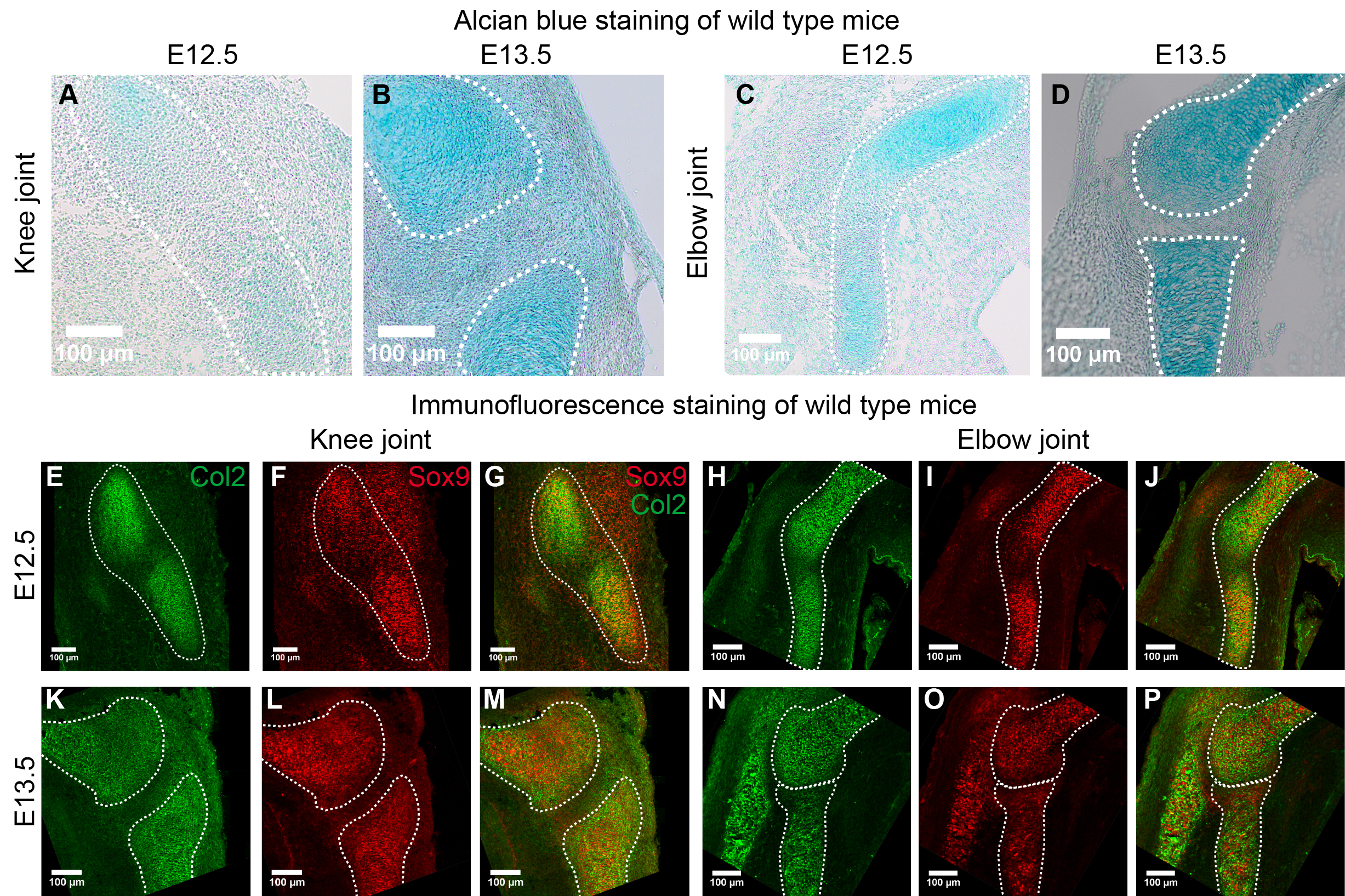

Supplement: FIGURE S1 — Alcian blue (A–D), Col2 and Sox9 (E–P) staining of the knee and elbow joint during at E12.5 and E13.5. The white dashed lines outline the cartilage. [file Image_1.jpeg]

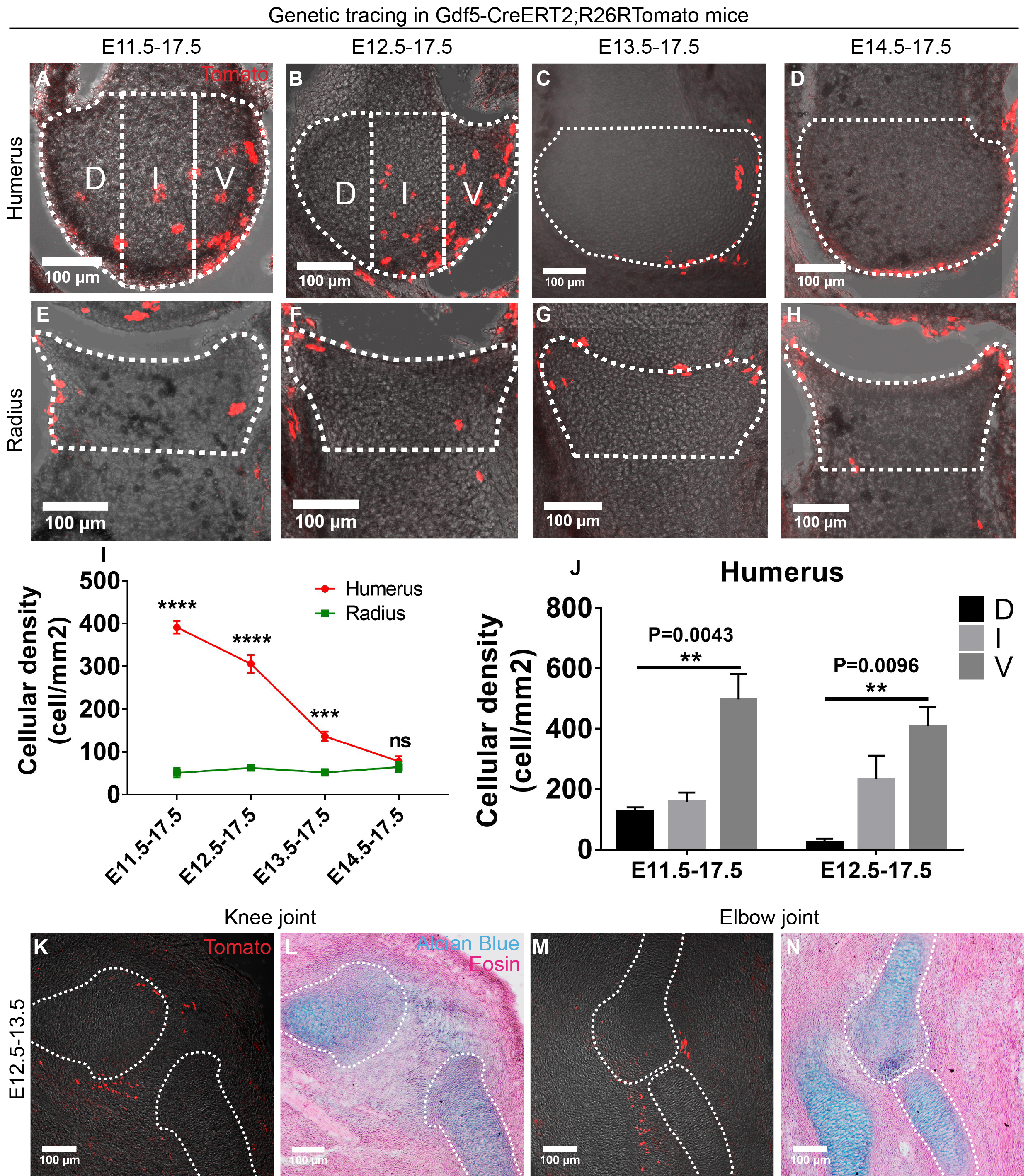

Supplement: FIGURE S2 — (A–H) Example images showing the distribution of traced Gdf5-expressing cells in the epiphyseal cartilage of elbow joint. D, I, and V refer to dorsal condyle, intercondylar eminence, and ventral condyle regions, respectively. (I) Quantification of the cellular density distribution among the four tracing periods revealed an asymmetrical contribution of the Gdf5-expressing cells to the epiphyseal cartilage of humerus and radius. (J) Quantification of the cellular density distribution in the D, I, and V regions during E11.5–E17.5 and E12.5–E17.5 tracings revealed an asymmetrical contribution of the Gdf5-expressing cells to the humerus epiphyseal cartilage along the dorsal-ventral axis. (K–N) 1-day tracings of the Gdf5-expressing cells from E12.5 to E13.5 in knee (K,L) and elbow (M,N) joints mainly labeled the cells around the joint forming region. (L) and (N) are alcian blue and eosin staining of the same tissue section on the left. Clonal density represents the number of traced clones over cartilage area (mm2). Data represent mean ± SEM, where at least five embryos were analyzed. The white dashed lines outline the epiphyseal cartilage. **P < 0.01, ***P < 0.005, ****P < 0.001. [file Image_2.jpeg]

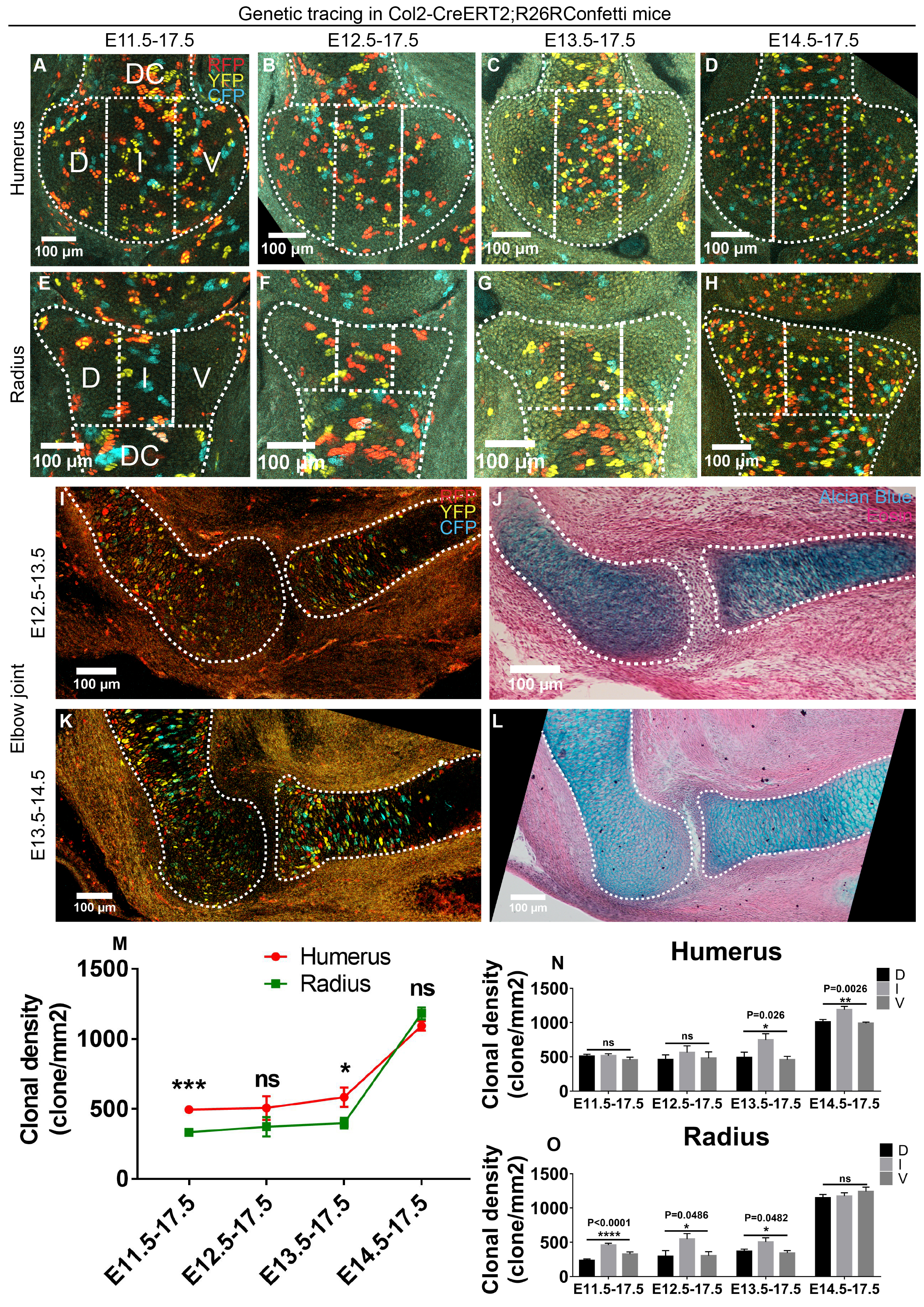

Supplement: FIGURE S3 — (A–H) Example images illustrating the distribution of traced Col2-expressing clones in the epiphyseal cartilage of elbow joint. D, I, and V refer to dorsal condyle, intercondylar eminence and ventral condyle regions, respectively. (I–L) 1-day tracings of the Col2-expressing cells from E12.5 to E13.5 and E13.5 to E14.5 mainly labeled the cells in the diaphyseal side of the cartilaginous anlagen. (J) and (L) are alcian blue and eosin staining of the same tissue section on the left. (M) Quantification of the clonal density distribution revealed an ascending trend in the epiphyseal cartilage among the four tracing periods. (N,O) Quantification of the clonal density distribution in the D, I, and V regions revealed an asymmetrical contribution of the Col2-expressing clones to the humerus and radius epiphyseal cartilage along the dorsal-ventral axis during many of the analyzed tracing periods. Clonal density represents the number of traced clones over cartilage area (mm2). Data represent mean ± SEM, where at least five embryos were analyzed. The white dashed lines outline the epiphyseal and diaphyseal cartilage (DC). *P < 0.05, **P < 0.01, ***P < 0.005, ****P < 0.001. [file Image_3.jpeg]

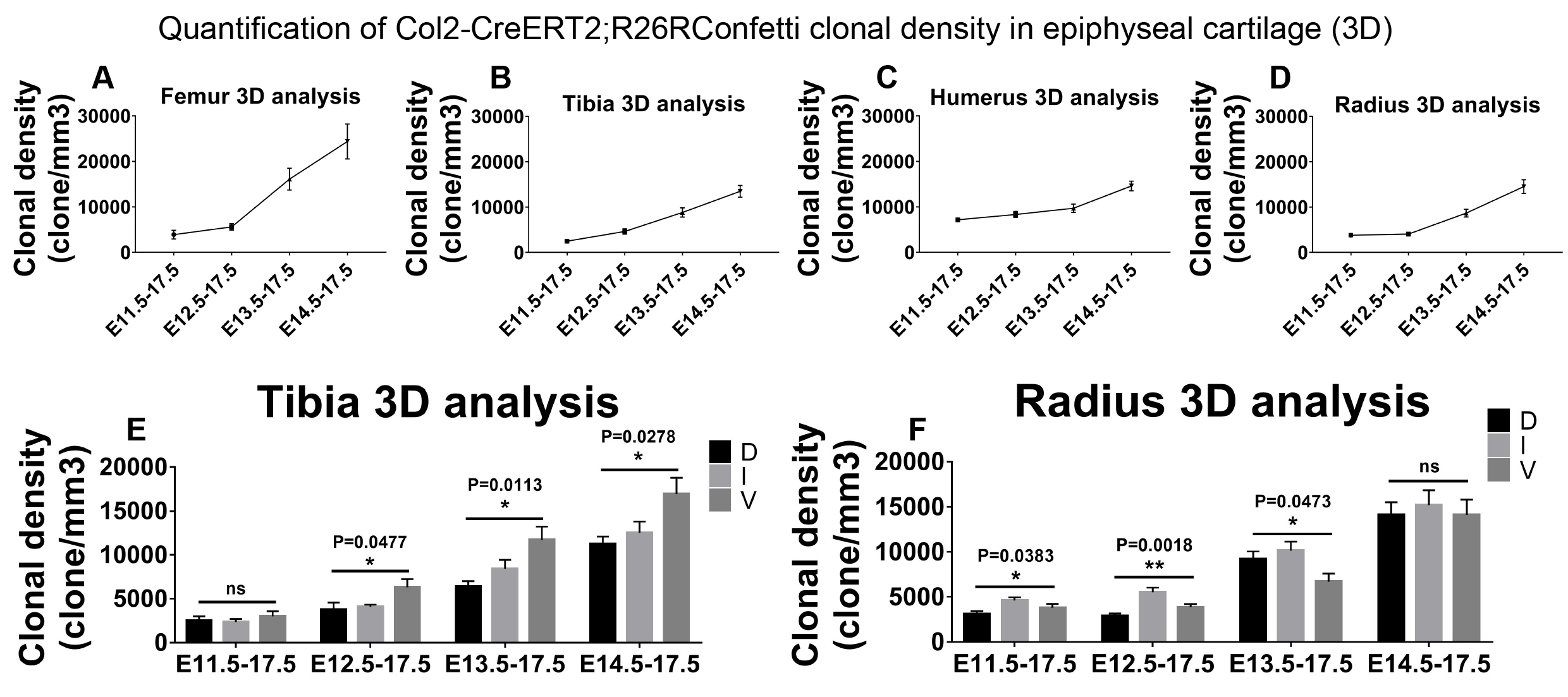

Supplement: FIGURE S4 — (A–D) 3D analysis of traced Col2-expressing clonal density in the entire epiphyseal cartilage of the four skeletal elements. (E,F) Distribution of traced Col2-expressing clones in dorsal condyle (D), intercondylar eminence (I), and ventral condyle (V) regions of the entire tibia and radius epiphyseal cartilage at the 3D level. Clonal density represents the number of traced clones over cartilage area (mm2). Data represent mean ± SEM, where at least five embryos were analyzed. *P < 0.05, **P < 0.01. [file Image_4.jpeg]

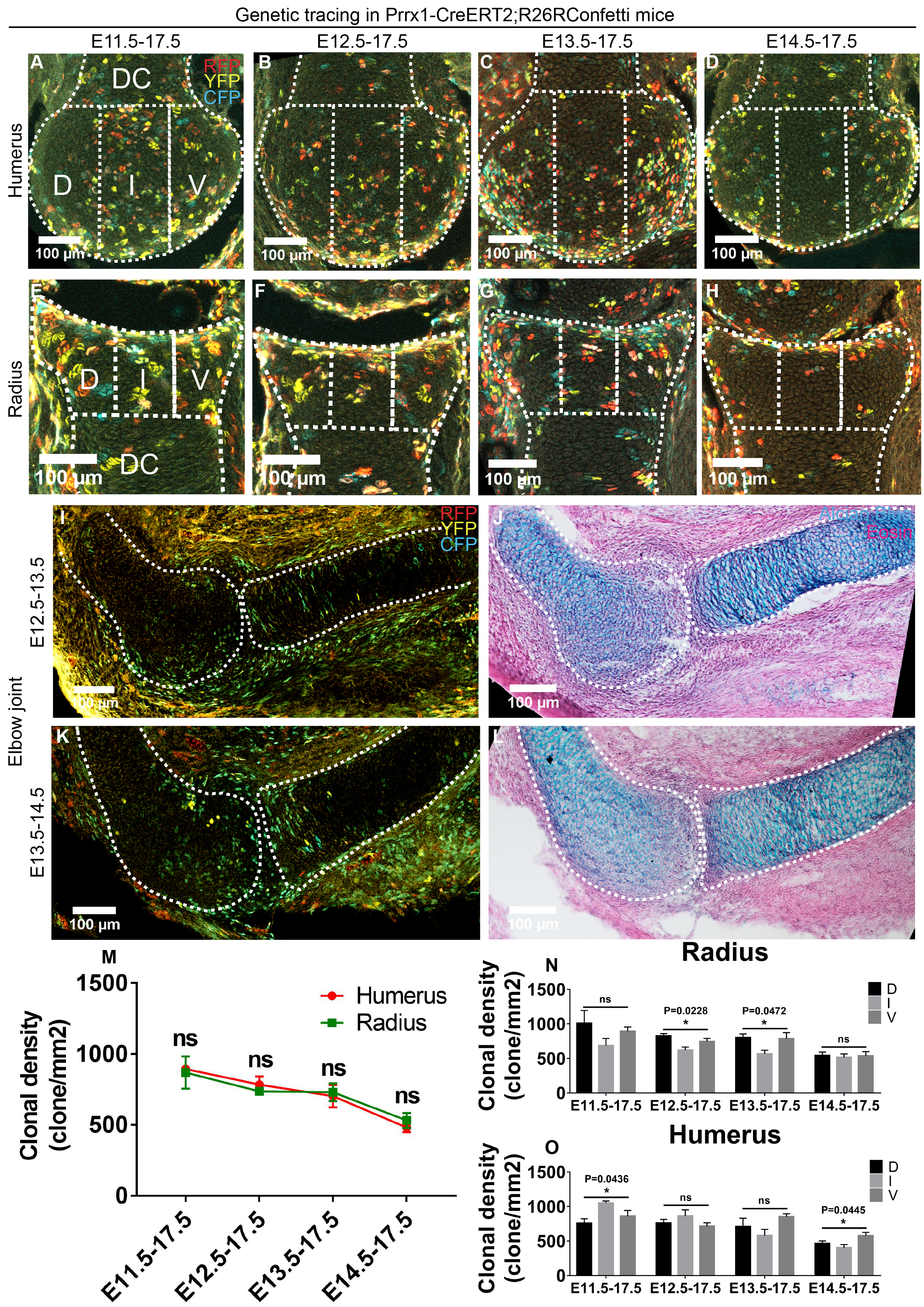

Supplement: FIGURE S5 — (A–H) Example images illustrating the distribution of traced Prrx1-expressing clones in the epiphyseal cartilage of elbow joint. D, I, and V refer to dorsal condyle, intercondylar eminence and ventral condyle regions, respectively. (I–L) 1-day tracings of the Prrx1-expressing cells from E12.5 to E13.5 and E13.5 to E14.5 mainly labeled the cells in the joint forming region and epiphyseal side of the cartilaginous anlagen. (J) and (L) are alcian blue and eosin staining of the same tissue section on the left. (M) Quantification of the clonal density distribution revealed a descending trend in the epiphyseal cartilage among the four tracing periods. (N,O) Quantification of the clonal density distribution in the D, I, and V regions revealed an asymmetrical contribution of the Prrx1-expressing clones to the humerus and radius epiphyseal cartilage along the dorsal-ventral axis during many of the analyzed tracing periods. Clonal density represents the number of traced clones over cartilage area (mm2). Data represent mean ± SEM, where at least five embryos were analyzed. The white dashed lines outline the epiphyseal and diaphyseal cartilage (DC). *P < 0.05. [file Image_5.jpeg]

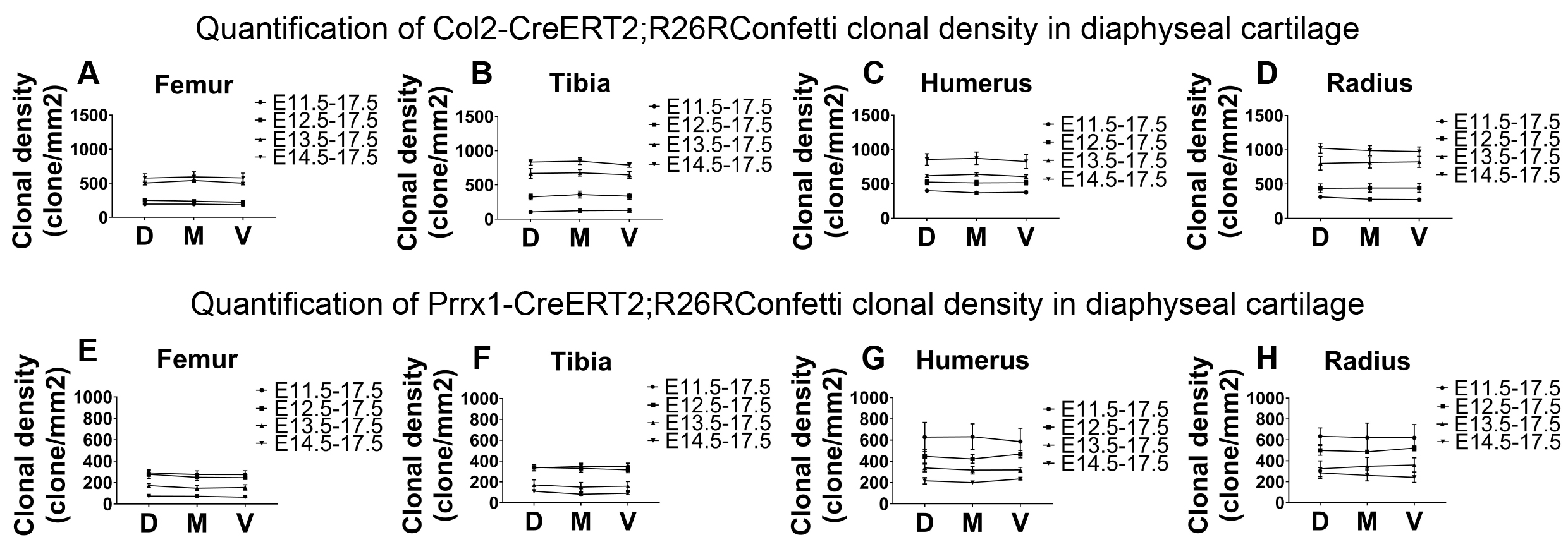

Supplement: FIGURE S6 — (A–H) The Col2- and Prrx1-traced clones distribute evenly in the dorsal condyle (D), intercondylar eminence (I), and ventral condyle (V) regions of the diaphyseal cartilage of the four skeletal elements. Clonal density represents the number of traced clones over cartilage area (mm2). Data represent mean ± SEM, where at least five embryos were analyzed. [file Image_6.jpeg]

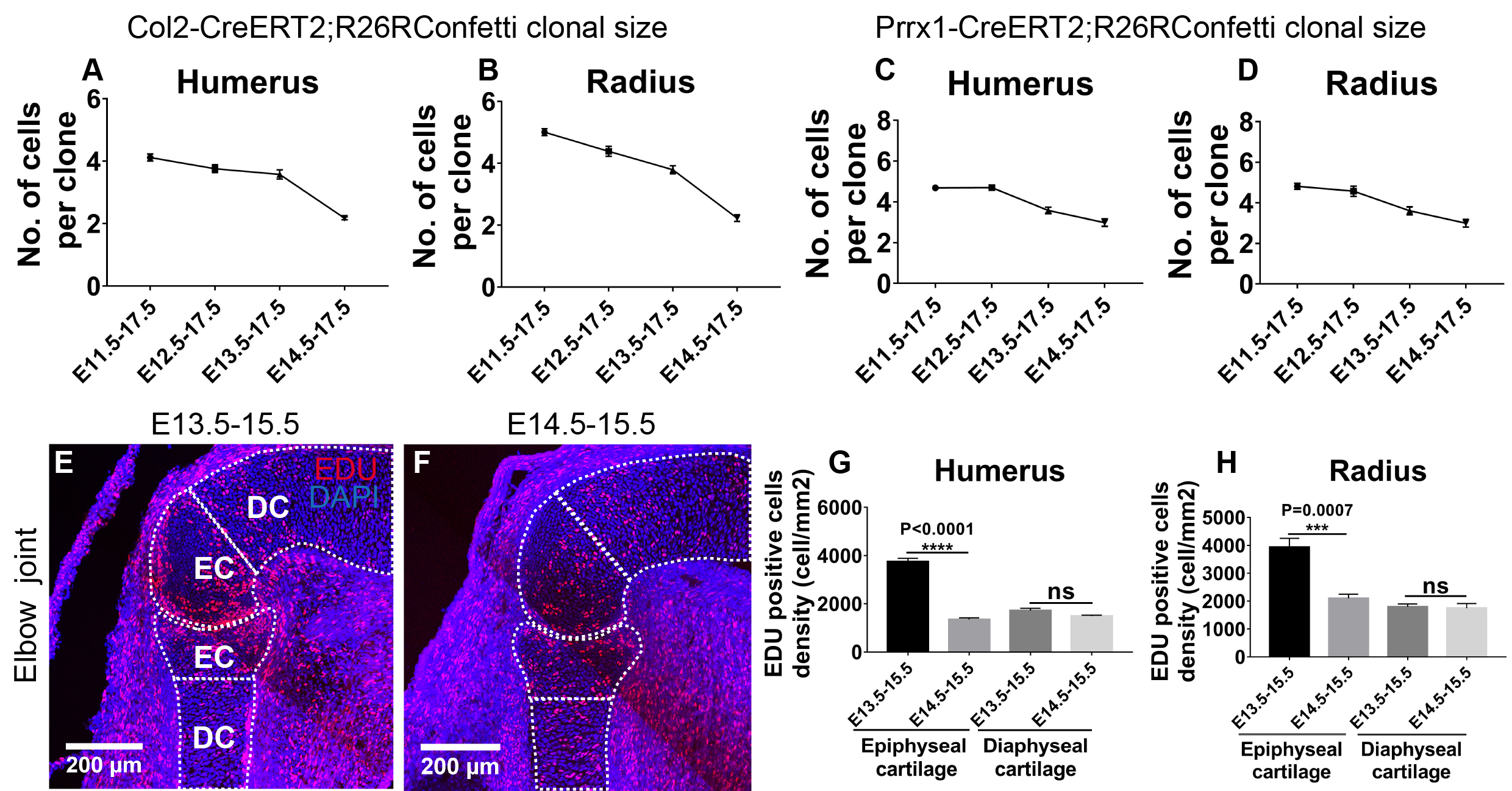

Supplement: FIGURE S7 — (A,B) Size of the traced Col2-expressing clones dropped sharply in the humerus and radius epiphyseal cartilage during E14.5–E17.5 tracing. (C,D) Size of the traced Prrx1-expressing clones gradually decreased as the tracing was initiated at later stages. (E,F) Example EDU staining images of elbow joint in wild type mice injected with EDU at E13.5 or E14.5. (G,H) More EDU-positive cells were found in the epiphyseal cartilage, but not the underneath growth plate, of both humerus and radius when EDU was injected at E13.5 compared to injection at E14.5. EDU-positive cell density represents the number of EDU-positive cells over cartilage area (mm2). Data represent mean ± SEM, where at least three embryos were analyzed. The white dashed lines outline the epiphyseal cartilage (EC) and diaphyseal cartilage (DC). ***P < 0.005, ****P < 0.001. [file Image_7.jpeg]

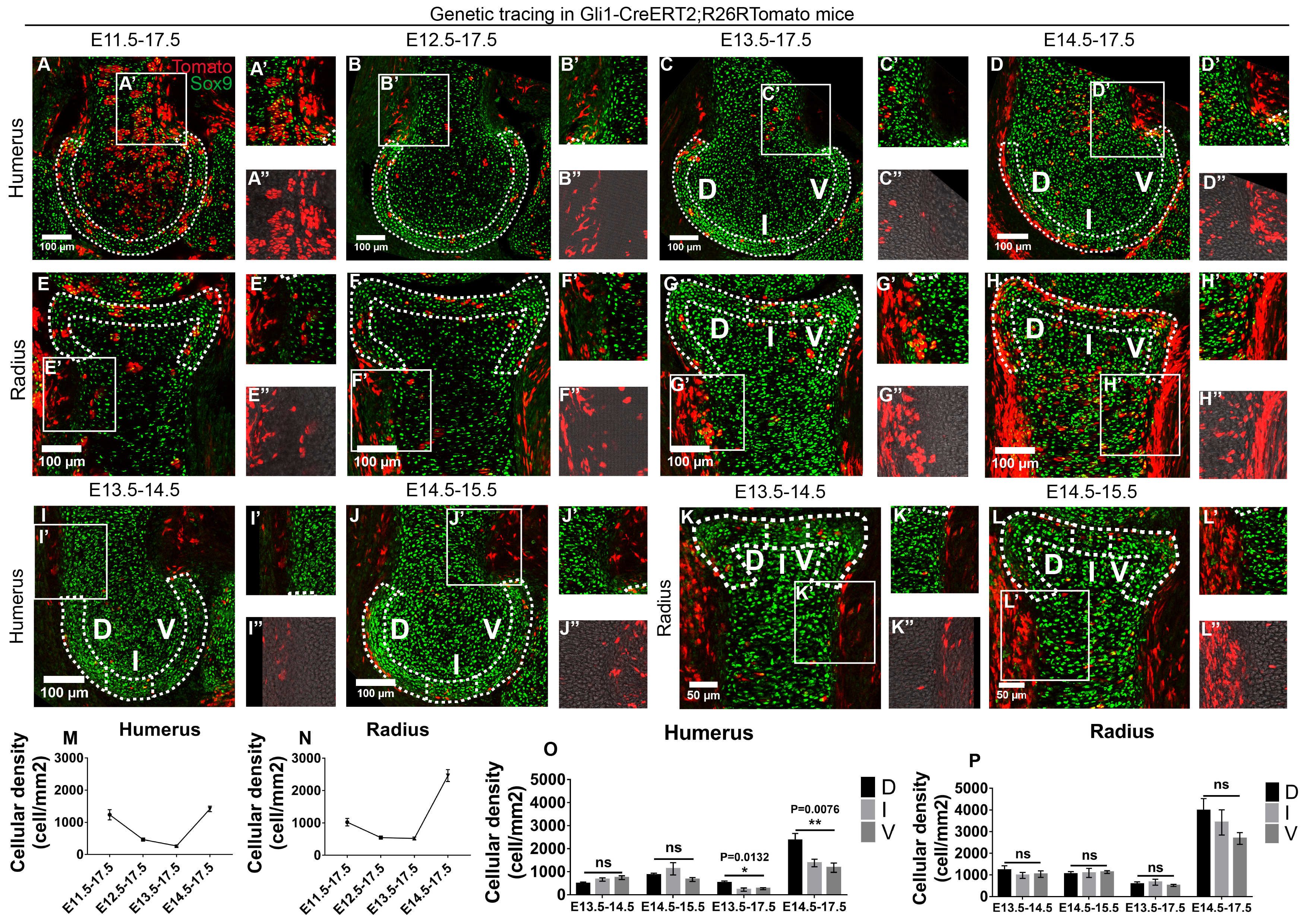

Supplement: FIGURE S8 — (A–L) Example images of lineage tracings in Gli1-CreERT2;R26RTomato mice at the elbow joint surface. (M–P) No obvious pattern was observed for Gli1-tracings at the elbow joint periarticular region. Data represent mean ± SEM where at least four embryos were analyzed. The white dashed lines outline the periarticular region of epiphyseal surface. D, I, and V refer to dorsal condyle, intercondylar eminence and ventral condyle sub-regions, respectively. *P < 0.05, **P < 0.01. [file Image_8.jpeg]

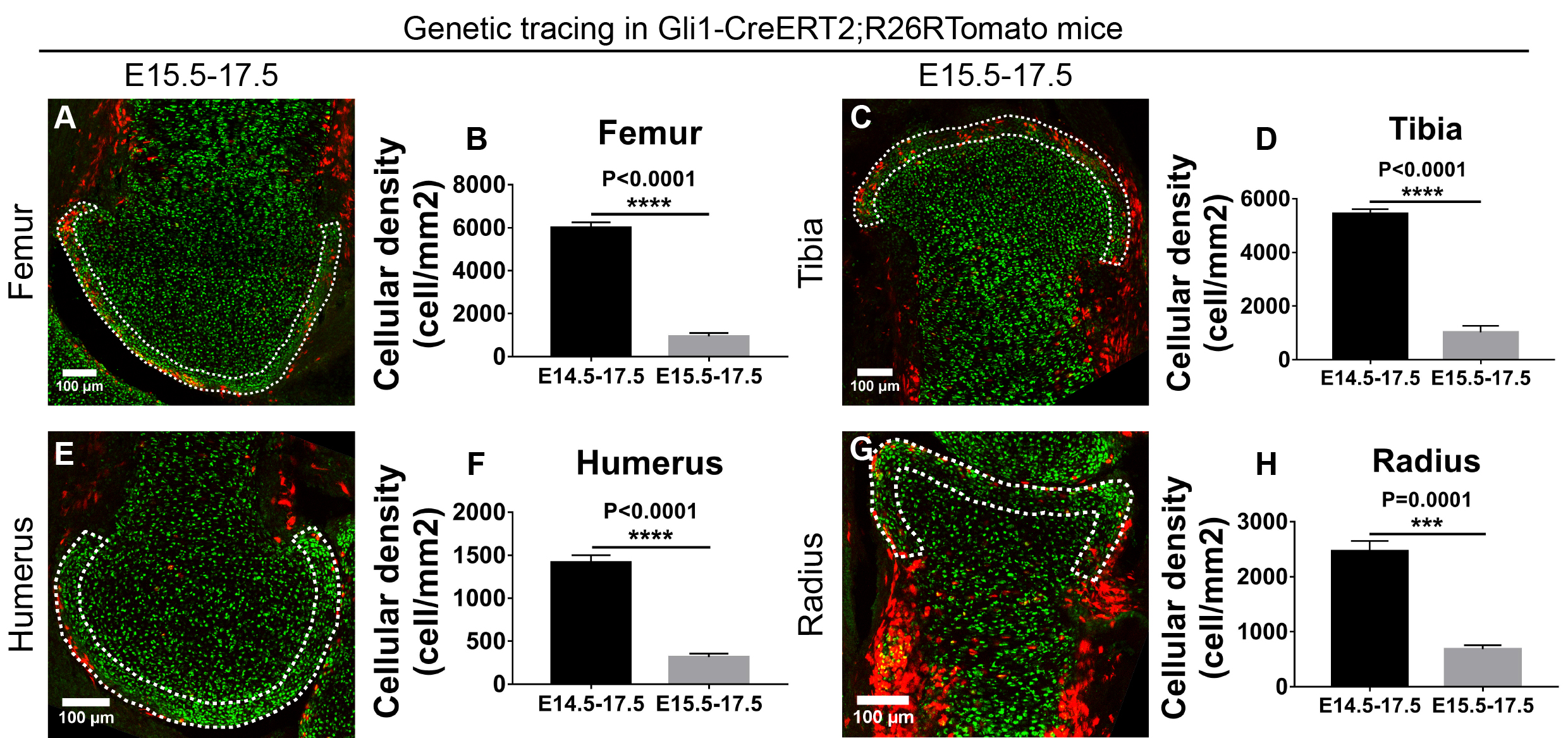

Supplement: FIGURE S9 — (A–H) Gli1-positive cell density dropped sharply in the periarticular region of epiphyseal surface of knee and elbow joint during E15.5–E17.5 tracing compared to E14.5–E17.5 tracing. Data represent mean ± SEM where at least four embryos were analyzed. The white dashed lines outline the periarticular region of epiphyseal surface. ***P < 0.005, ****P < 0.001. [file Image_9.jpeg]
